# Cerebrospinal Fluid Leak in Cochlear Implantation: Enlarged Cochlear versus Enlarged Vestibular Aqueduct (Common Cavity Excluded)

**DOI:** 10.1155/2016/6591684

**Published:** 2016-10-26

**Authors:** Giovanni Bianchin, Valeria Polizzi, Patrizia Formigoni, Carmela Russo, Lorenzo Tribi

**Affiliations:** Otorhinolaryngology and Audiology Department, ASMN-IRCCS Hospital, Reggio Emilia, Italy

## Abstract

*Objective*. To share our experience of cerebrospinal fluid gusher in cochlear implantation in patients with enlarged cochlear or vestibular aqueduct.* Study Design*. Case series with comparison and a review of the literature.* Methods*. A retrospective study was performed. Demographic and radiological results of patients with enlarged cochlear aqueduct or enlarged vestibular aqueduct in 278 consecutive cochlear implant recipients, including children and adults, were evaluated between January 2000 and December 2015.* Results*. Six patients with enlarged cochlear aqueduct and eight patients with enlarged vestibular aqueduct were identified. Cerebrospinal fluid gusher occurs in five subjects with enlarged cochlear aqueduct and in only one case of enlarged vestibular aqueduct.* Conclusion*. Based on these findings, enlarged cochlear aqueduct may be the best risk predictor of cerebrospinal fluid gusher at cochleostomy during cochlear implant surgery despite enlarged vestibular aqueduct.

## 1. Introduction 

In the last few decades, advances in cochlear implant (CI) technology and technique have resulted in improved surgical outcomes in subjects with cochlear malformations [1]. Knowledge of the fine structures of the temporal bone using high resolution CT is essential for correct planning of surgery, preventing surgical complications, and predicting the outcome of the procedure. A minimum anatomical requirement for CI is the presence of an implantable cavity near stimulable elements whose projections connect to the auditory cortex [2].

The reported prevalence of inner ear malformation in individuals with congenital deafness or sensorineural hearing loss varies widely from 2.3% to 28.4% depending on patient selection criteria [3].

According to the literature, all cochlear dysplasias can be implanted, with the exception of cochlear aplasia. Another absolute contraindication is the absence of the acoustic nerve [4].

Recent reports of experience with implantation of children and adults with cochlear abnormalities have demonstrated that implantation results in levels of performance not unlike that seen in patients with normal anatomy. Nevertheless cochlear malformations may occur with a higher percentage of surgical complications [5].

Intraoperative cerebrospinal fluid (CSF) leakages from the cochleostomy site are a serious complication of cochlear implantations. They occur as a result of abnormal communication between CSF and perilymph in the cochlea with possibility of residual CSF fistula and a hypothetic increased risk for developing meningitis postoperatively [6].

The incidence of CSF leak in cochlear implantation is reported to be between 1 and 5% in large case series [7–10].

In 1987, Jackler et al. classified cochlear malformations into five types: complete labyrinthine aplasia, cochlear aplasia, cochlear hypoplasia, incomplete partition, and common cavity [11]. Sennaroglu and Saatci proposed a new classification system in 2002 that distinguished incomplete partition into two types: cystic cochleovestibular malformation (IP-I) and the classic Mondini deformity (IP-II) [4].

However, the degree of dysplasia is not necessarily correlated to the risk of CSF leakage [12, 13].

The pathogenesis of perilymph fistulas is thought to be the result of direct communication between the subarachnoid space and inner ear caused by a defect in the bony partition of the fundus of the internal auditory canal (IAC) [13, 14]; enlargement of the cochlear aqueduct (ECA) and vestibular aqueduct (EVA) has also been suggested as a cause of sensorineural hearing loss and perilymph fistula [15].

**Figure 1:**
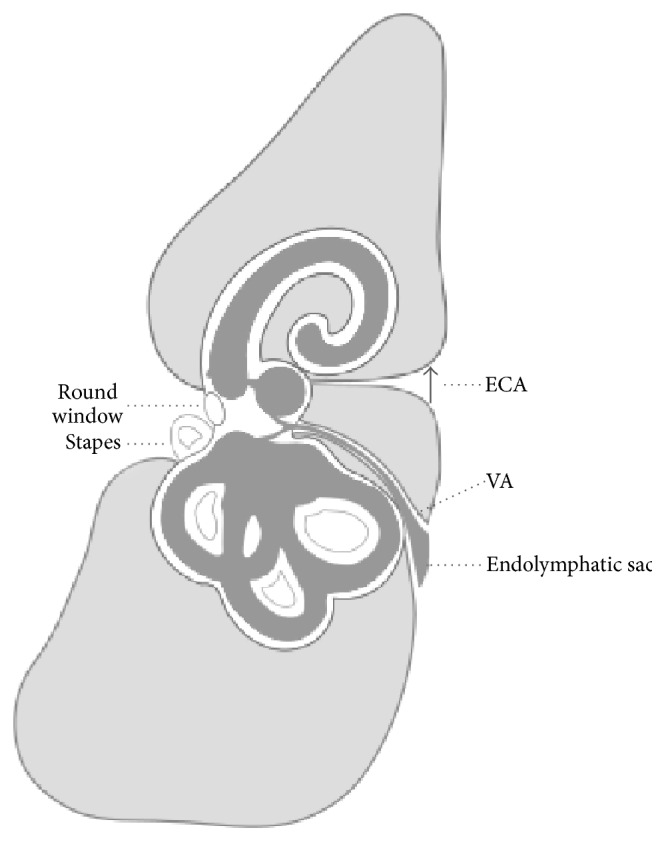
Enlarged cochlear aqueduct (ECA) and vestibular aqueduct (VA).

The preoperative CT and MRI of the internal auditory canal do not make it currently possible to check the conditions of the fundus of the internal auditory canal and, in particular, an abnormal connection between the arachnoid space and inner ear. This condition can only be suspected, there are no dedicated studies, and the aetiological hypothesis is made after other possible causes have been ruled out. It is, however, different for the ECA and EVA studies; these can easily be conducted radiologically by means of current CT methods. Evaluating the conditions of these aqueducts which can provoke CSF leak may be particularly useful in planning cochlear implantation surgery, stapedectomy, and others (Figure 1) [16].

*Anatomy and Physiology of Cochlear Aqueduct*. The CA is a small bony canal in the temporal bone that connects the subarachnoid space of the posterior fossa to the basal turn of the cochlea [17]. Generally, it is located 7 mm below the internal acoustic meatus and at the upper edge of the jugular foramen [18].

The lumen of the CA is elliptic, and its largest diameter lies in the horizontal plane [19]. It contains the perilymphatic duct, which connects the subarachnoid space with the scala tympani and is filled with a loose mesh of connective tissue that, although permeable to fluid, limits the patency of the CA [13]. For the most part, it is filled by loose fibrous connective tissue, which is similar to that surrounding the endolymphatic sac. This layer of connective tissue is continuous laterally with the endosteal covering of the scala tympani and medially with the dura and arachnoid, which extends into the canal to a variable length. Therefore, unlike the vestibular aqueduct, the cochlear aqueduct does not contain a true epithelium-lined duct [20].

The CA runs a downward oblique course between the cochlea and the subarachnoid space. Thanks to the CT the course of the CA is divided into four segments [13].

The* lateral orifice *is the narrow opening of the bony aqueduct into the basal turn of the cochlea. It is located along the anteroinferior edge of the scala tympani immediately anterior to the crest of the attachment of the round window [21, 22]. The lateral orifice opens into the* otic capsule segment*, which runs medially through the labyrinthine bone. The otic capsule segment becomes continuous with the* petrous apex segment*, medially. The petrous apex segment runs through bone, which may be either pneumatized or filled with marrow. The petrous apex segment opens into the subarachnoid space adjacent to the pars nervosa of the jugular foramen via the tunnel-shaped* medial orifice* [13–23].

A classification of CA types was proposed by Migirov and Kronenberg, 2005, A type 1 CA was wholly visualized from the cochlea to the medial (external) aperture; a* type 2* CA was detected in the medial two-thirds of the duct; a* type 3* CA was only observed in the external aperture and/or the medial third; and a* type 4* CA was not visible in whole CT images [23].

The CA provides a conduit between the posterior fossa and the inner ear, thereby permitting transmission of perilymphatic fluid, which originates partly from cerebrospinal fluid, into the inner ear [24, 25].

This anatomical structure is believed to be involved in controlling the pressure of the perilymphatic fluid (PF).

The contents of the PF and CSF are similar, except for some difference in electrolyte concentration; the perilymph possibly originates from the CSF [26].

The clinical significance of CA remains unknown, and the effect of an enlarged CA is controversial. The fact that a patent cochlear aqueduct provides a channel of communication between the subarachnoid space and the perilymph has been established experimentally in laboratory animals [27].

The cochlear aqueduct may function as a pressure-regulating mechanism equalizing CSF pressure with perilymph pressure [28, 29], an attractive hypothesis similar to that proposed for the endolymphatic sac, but this has not been demonstrated in humans.

The narrow diameter of the CA is thought to buffer the inner ear from the wide pressure variations present within the posterior fossa subarachnoid spaces [15, 26, 30].

CA is supposed to filter out cardiac and respiration induced pulses in CSF and prevents them from affecting cochlear function. Decreasing cerebrospinal fluid pressure, for example, after lumbar puncture, can lead to sensorineural hearing loss. This clinical phenomenon is declared by the following hypothesis: lumbar puncture causes volume loss in the CSF-space with consecutive decrease of pressure in this compartment. CSF-space communicates with the perilymph space via the cochlear aqueduct, leading to a pressure gradient between the perilymph space and the CSF-space, causing transport of perilymphatic fluid into the cerebrospinal subarachnoid space. The consequence is loss of volume in the perilymph space and an imbalance of pressure on the border between perilymph and endolymph. This could result in compensatory expansion or hydrops of the endolymphatic space [31, 32].

The CA can be a difficult anatomical structure to visualize in radiological studies.

Evaluation of the normal CT appearance of the cochlear aqueduct in high resolution studies (1 mm thick sections) showed that the CA diameter was dependent on the specific segment being studied. This inability to see portions of the cochlear aqueduct is most likely due to its small diameter, which, in anatomic studies, has been reported to be between 0.1 and 0.2 mm at its midportion [33, 34]. For Gopen et al. the average mean diameter of the midotic segment of the visualized aqueducts was 0.56–0.26 mm [18]. The most visible portion of the CA is the medial orifice, which was identified in 97% of patients and has a mean diameter of 1.76 ± 0.87 mm [35]. The median aperture is highly variable, ranging from 2 up to 4 mm; there was no age correlation. This dimension is considerably larger than that measured by Palva and Dammert (1.8 mm) [19] and Anson et al. (0.8 mm) [20].

**Figure 2:**
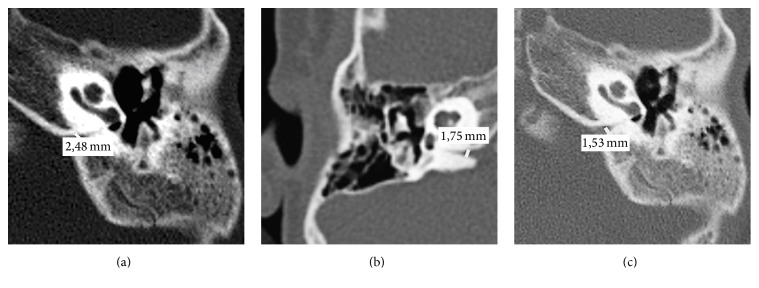
Enlarged cochlear aqueduct.

CA enlargement has been defined with a diameter of more than 1 mm in the entire otic capsule segment (Figure 2) [23].

Probably the measurement of the width of the median orifice may be taken as a criterion for distinguishing a normal cochlear aqueduct from an enlarged one since it is better visible under CT, but its limit depends on the vast variability of its diameter in the normal population.

It is not possible to correlate the ECA with a precise syndrome; it may occasionally be associated with other internal ear anomalies. There are no studies in literature that correlate the presence of ECA with congenital hearing loss nor does there seem to be a risk factor for acquired sensorineural hearing loss.

ECA may play a role in the pathogenesis of congenital hearing loss and in the risk of perilymphatic gusher following stapes surgery and cochleostomy during cochlear implant.

*Anatomy and Physiology of Vestibular Aqueduct*. The VA is the bony canal containing the endolymphatic duct connecting the endolymphatic sac and the vestibule [36].

The vestibular aqueduct drains endolymph and connects the membranous labyrinth (utricle and saccule) with the endolymphatic sac.

The function of the endolymphatic duct and sac is not totally understood, but it is believed that they help maintain the volume and ionic composition of endolymph necessary for transmitting hearing and balance nerve signals to the brain.

It has an average diameter of 0.6 to 1.5 mm at its midpoint between the common crus and its opening at the posterior cranial fossa. Measuring approximately 10 mm long, it starts at the medial wall of the vestibule and stretches posteriorly before opening in the petrous pyramid.

Inside the VA is the endolymphatic duct, a tube that connects the endolymph (a fluid) in the inner ear to the endolymphatic sac [37].

**Figure 3:**
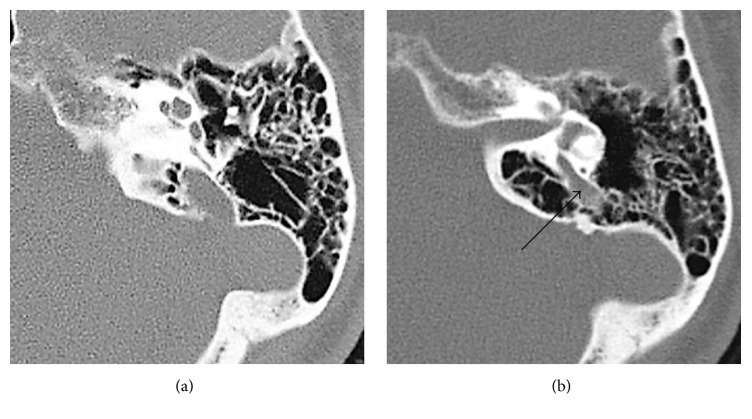
Enlarged vestibular aqueduct.

EVA has been found to be the most common inner ear malformation associated with sensorineural hearing loss. It may be seen as part of the Mondini anomaly [38] but it may also be seen as an isolated entity. It is commonly seen as a feature of Pendred syndrome (Figure 3) [39–41].

In the Nottingham Paediatric Cochlear Implant Programme 4% CI candidates were identified as having EVA [42].

Recently, with MRI and CT imaging, the incidence of other inner abnormalities associated with EVA has been reported to range from 41% to 88% [43].

In 1978, Valvassori and Clemis were the first to use imaging. They examined 3700 patients, reporting 50 cases with EVA (i.e., 1.5%), observing an association between enlargement of the vestibular aqueduct and neurosensory hypoacusis. The association of this anatomical anomaly with sensorineural hearing loss led them to coin the term “enlarged vestibular aqueduct syndrome” (EVAS). 50% of subjects with EVA were children and adolescents (female/male 3 : 2; bilateral/unilateral 2 : 1) [36].

EVA is usually bilateral. Genetic testing often but not always reveals that EVA is associated with mutation of the SLC26A4 gene.

Mutations of the pendrin SLC26A4 gene are considered to be one of the most common causes of congenital hearing loss and EVA and are involved in about 10% of all hereditary hearing losses.

Two clinical manifestations are associated with mutations of the pendrin gene: EVA, hypothyroidism, and hearing loss (which express the Pendred syndrome) and a nonsyndromic form with hearing loss and EVA. Pendred syndrome occurs in an estimated one-third of all cases of EVA [44] and is an autosomal recessive genetic disorder, meaning each parent must be a genetic carrier [45].

Furthermore, EVA may also be associated with syndromic hearing loss as well as in Pendred syndrome, branchiootorenal syndrome and CHARGE syndrome (coloboma of the eye, heart defects, atresia of the nasal choanae, retardation of growth and/or development, genital and/or urinary abnormalities, and ear abnormalities and deafness). It has been postulated that EVA is inherited as an autosomal recessive trait [46].

In a proportion of patients with EVA, a sudden drop in hearing can even occur especially after a minor head trauma.

A child with EVA will hear normally in the first years of life and then notice hearing loss later in childhood or less commonly in adolescence or early adulthood. Generally, this occurs after a minor or major head impact, upper respiratory infection, or air pressure trauma, such as that which occurs during the rapid depressurization of an airplane [47].

## 2. Materials and Methods

Cases of CI subjects with bilateral profound deafness with ECA and EVA were examined. The cases of common cavity were excluded. CC was defined as the absence of internal differentiation into either a vestibular or cochlear bud.

The cases were identified among the case histories over the last 15 years (from January 2000 to December 2015) at the ORL Division of the Arcispedale Santa Maria Nuova of Reggio Emilia consisting of 278 cases of cochlear implant. This time window was intentionally selected in which the most modern CT technique was used which improves the study of these thin structures. The cases were separated into two groups: one with 6 patients (5 children with average age 17 months, range 1.1 y to 1.5 y, and 1 adult aged 63 years) with ECA and the other with 8 patients (6 children with average age 16 months, range 1 y–1.8 y, and 2 adults aged 56 y and 63 y) for EVA.

ECA is defined as an aqueduct which under CT can be recognised throughout its length (all the segments are visible) and a diameter of more than 1 mm in the medial portion [23].

EVA is defined by the criteria originally established by Valvassori and Clemis [36] and further revised in recent years by the Cincinnati group [48].

A VA diameter greater than or equal to 1.5 mm in the median point or greater than or equal to 2 mm for the operculum was defined as an EVA.

 A central point of 1.0–1.4 mm and 1.5 to 1.9 mm operculum were considered as borderline EVA.
 Less than 1.0 mm for the median point and 1.5 mm for the operculum were reported as normal VA [38, 49–51].

All patients underwent a successful transmastoid intact canal wall approach to CI with insertion of multicanal electrodes by means of cochleostomy at basal turn. The round window access was never used.

The operators' reports containing the description with the presence/absence of the CSF gusher at cochleostomy were examined. The term gusher is generally used in literature to describe the egress of profuse clear fluid when an opening is made into the inner ear [5, 52].

It was considered as gusher only in those who had profuse CSF outflow upon opening the cochlea which usually lasts several minutes. A gentle flow of clear fluid is called “oozing” and a profuse flow is termed “gusher” [5, 53].

Cases with pulsatile perilymph, oozing, or leaks that are sometimes found on opening the cochlea were not enrolled in this study [12].

The surgeon always waits for several minutes until the gusher slows down considerably before attempting electrode insertion. All the CSF were controlled by the application of the electrode and obliteration of the cochleostomy with muscle layers and fibrin glue. In all patients, a watertight seal was created around the cochlear implant array by means of a customized technique using four to five pieces of fresh muscle.

## 3. Results 

The results are described in Table 1.

For the enlarged cochlear aqueduct there was gusher in 5 cases. In one case there was no leakage of perilymph at cochleostomy.

For the enlarged vestibular aqueduct, there was gusher in only one case, while in 7 other cases there was no leakage of perilymph on cochleostomy.

## 4. Discussion 

The development of the CA and its adjacent structures in the human fetus occurs over a 6-week period [54, 55]. At 22 weeks of gestation, the petrous apex ossifies and forms the medial wall of the CA, and the progressive otic capsule ossification leads to the formation of the lateral wall of the CA [56, 57]. Subsequently, the width of the CA diminishes with gestational age. ECA may be the result of arrest in temporal bone embryogenesis. Therefore, an enlarged CA may result from the developmental arrest of the primitive CA [58].

The dilated canal can be explained by a developmental arrest in the embryogenesis of the primitive cochlear aqueduct and its embryonic contents [19]. Histological findings report that the VA is wider in the initial weeks of gestation and decreases progressively with the development of the temporal bone until the final dimensions are reached [58]. The VA derives from a diverticulum formed in the wall of the otocyst during the fifth week. The aqueduct begins as a short, broad pouch but gradually elongates and thins until it achieves its characteristic J shape of adulthood.

EVA may be the result of arrest of embryological development at the early 5-week stage [59]. Many theories have been proposed on the formation of EVA, but their cause remains unknown.

There are two general theories regarding the origin or cause of EVA.

- Jackler and De La Cruz suggest that a stop in development of the VA in the fifth week of gestation results in an EVA due to failure of shrinking of the structure [59].
- Other authors believe that EVA is the result of aberrant delayed development of the conduit and saccule in foetal and postnatal life, while, for other authors, it is the result of a continuous growth rather than stop in the development of embryogenesis [60].

The CSF leak, the so-called gusher, is a frequent eventuality in ear surgery meant for CI and for otosclerosis surgery.

Intraoperative CSF leakages from the cochleostomy site are a serious complication of cochlear implantation surgery [61].

Various techniques to control the CSF leak at cochleostomy have been described and can be utilized according to the severity of the leak. We used some techniques to control CSF gusher leak like tight sealing of the insertion site, sealing the insertion site with temporalis muscle fascia graft around the electrode array and tissue glue. Literature contains a description of the elevation of the head end of the table to control CSF leak. We do not place the patient in the reverse Trendelenburg position to slow the flow of CSF. We believe that this may make the CSF flow deceptively slow so one cannot be sure whether packing was effective or not. Some authors suggest a lumbar drain to control colostomy gusher. Loundon et al. suggested osmotherapy as an effective means for control of leakage during cochleostomy [62].

The pathogenesis of perilymph fistulas is thought to be the result of a direct communication between the subarachnoid space and inner ear caused by a defect in the bony partition of the fundus of the IAC [14, 15] (not identifiable with studies of images or other methods) or enlarged cochlear or vestibular aqueduct [13, 16, 32]. For a long time, EVA was considered as the main cause of the presence of intraoperative CSF leak [63, 64].

The incidence rate of CSF gushers in CI patients with EVA according to some authors was approximately 4.8% [9]. Other authors reported that approximately 5% of cases result in CSF gushers, primarily among patients with inner ear malformations and especially those with a Mondini malformation [65]. Other authors found different contradicting data. Miyamoto et al. report on a series of patients with enlarged vestibular aqueduct who underwent CI. Surgery was without complication although there were reports of pulsatile clear fluid arising from the cochleostomy in five patients (total of 14 adults and 9 children) [66].

No gusher was experienced by Harker et al. in cochlear implantation in five EVA patients [67].

Our study too brings to light the fact that the CSF leak is not supported by the dilation of the vestibular aqueduct. On the other hand, there appears to be a direct correlation with the presence of enlarged cochlear aqueduct. In spite of the few cases, owing to the low incidence of the pathology, the correlation is 0.01% according to Visvanathan and Morrissey [68]. The use of the same electrode insertion technique (cochleostomy at basal turn) makes it possible to compare the cases correctly and use the same assessment for the presence of CSF leak. The presence of gusher in cases with ECA is 84%, a datum highly significant especially compared with the 12.5% of cases of EVA. Moreover, inclusion of the malformation called “common cavity” eliminates an important variable, making the study group highly selective and rigorous.

From the physiopathological point of view, the justification of the clear incidence of CSF leak in cases with ECA may be due to the fact that CA places in direct communication the subarachnoid space with the basal turn of the cochlea thereby allowing the backflow of the perilymph. One of its anomalies may be the basis of a malfunctioning of the perilymph drainage mechanisms and may therefore be responsible for a greater probability of encountering gusher during the cochleostomy procedure in operations involved in positioning the cochlear implant because of its direct action on the perilymph. However, the vestibular aqueduct contains the endolymphatic duct which is found to be formed of the union of the outlet openings of the utricular and saccular branches of the duct and which ends in the endolymphatic sac (a sort of intracranial extension of the membranous labyrinth) and through the dura mater contracts in relation to the sigmoid sinus and the cerebellum. The endolymphatic sac carries out the function of reabsorption of the endolymph produced by the vascular streak of the membranous cochlea and its action is therefore expressed on the endolymph.

The number of cases is limited, as already mentioned; therefore other correlations are hardly statistically significant. Similarly it is reported that controlling the leakage of liquor was not particularly difficult and there were no major complications in the postoperative phase but only minor problems characterised by neurovegetative disorders.

## 5. Conclusion

Our evaluation, based on a well selected group of cases and with a small number of cases, considering the rarity of the pathology, shows how, in comparison between the two enlarged aqueducts, the enlargement of the cochlear aqueduct causes gusher while the presence of EVA has lesser significance.

## Figures and Tables

**Table 1:** Features of ECA and EVA subjects.

	Age at implantation	Sex	Other temporal bone anomalies	Intraoperative complications	Electrode insertion approach to cochlear implantation	Postoperative complications
ECA						
1	16 m	M	ECA only	None	Complete cochleostomy	None
2	18 m	F	ECA only	CSF gusher	Complete cochleostomy	Nausea, vomit
3	13 m	F	Incomplete partition (type 1)	CSF gusher	Complete cochleostomy	Nausea, vomit
4	22 m	M	Incomplete partition (type 1)	CSF gusher	Complete cochleostomy	Nausea, vomit
5	18 m	M	Incomplete partition (type 1)	CSF gusher	Complete cochleostomy	None
6	63 y	F	ECA only	CSF gusher	Complete cochleostomy	Nausea, vomit, dizziness
EVA						
1	18 m	M	EVA only	None	Complete cochleostomy	None
2	12 m	F	EVA only	None	Complete cochleostomy	None
3	15 m	F	Incomplete partition (type 2)	CSF gusher	Complete cochleostomy	Nausea, vomit
4	14 m	M	EVA only	None	Complete cochleostomy	None
5	16 m	M	Semicircular canals dysplasia	None	Complete cochleostomy	None
6	21 m	F	Semicircular canals dysplasia	None	Complete cochleostomy	None
7	57 y	M	EVA only	None	Complete cochleostomy	Dizziness
8	63 y	F	EVA only	None	Complete cochleostomy	None
